# Batai Orthobunyavirus: An Emerging Mosquito-Borne Virus in Europe

**DOI:** 10.3390/v14091868

**Published:** 2022-08-25

**Authors:** Karen L. Mansfield, Arran J. Folly, Luis M. Hernández-Triana, Sanam Sewgobind, Nicholas Johnson

**Affiliations:** Animal and Plant Health Agency (APHA), Addlestone KT15 3NB, UK

**Keywords:** Čalovo, Chittoor, emerging infectious diseases, zoonotic

## Abstract

Batai virus (BATV) is a zoonotic orthobunyavirus transmitted by a wide range of mosquito vectors. The virus is distributed throughout Asia and parts of Africa and has been sporadically detected in several European countries. There is increasing evidence that BATV is emerging in Europe as a potential threat to both animal and human health, having been detected in mosquitoes, mammals, birds and humans. In recent years, serological surveillance in cattle, sheep and goats has suggested an antibody prevalence of up to 46% in European livestock, although human serological prevalence remains generally low. However, the recent and continued spread of invasive mosquito species into Europe may facilitate the establishment of competent populations of mosquitoes leading to increased BATV transmission. Migratory birds may also potentially facilitate the emergence of BATV in geographical locations where it was previously undetected. Although BATV has the potential to cause disease in humans and livestock, our understanding of the impact in wild animal populations is extremely limited. Therefore, there is a need for increased surveillance for BATV in mosquitoes, livestock, wild mammals and birds in Europe to understand the true impact of this virus.

## 1. Introduction

Batai virus (BATV) was first detected in Malaysia in 1955 [1,2]. Since then, it has been periodically detected across three continents, Asia, Europe and Africa, and from a range of mosquito, mammalian and avian species. It has never been detected in North or South America, although there has been an isolated detection in Australia [3]. BATV is thought to be transmitted in a vertebrate–zoophilic mosquito cycle [1], and there is strong evidence that it infects both humans and ruminants [1,4,5,6,7]. However, there is no evidence of animal-to-animal transmission.

BATV was first detected in *Culex gelidus* mosquitoes trapped in Malaysia in 1955 near the village of Batai just west of the Malaysian capital, Kuala Lumpur [1,2]. It was subsequently detected in *Anopheles maculipennis* from South Slovakia in 1960 [8], where the isolated virus was initially termed Čalovo virus but was subsequently shown to be genetically identical to BATV [9]. In 1971, the ‘Olyka’ strain of BATV was isolated in West Ukraine [10,11]. Another variant, termed Chittoor virus, has been detected in India in *An. barbirostris* [12]. Further isolates of BATV in Asia have been detected in southern Japan in bovine blood [13] and *An. philippines* (current name *An. phillippinensis*) in China [5]. There have been subsequent isolations of BATV from a range of mosquito species in several European countries, including *An. maculipennis* complex mosquitoes in Italy [14] and Germany [15].

Batai orthobunyavirus (BATV) is classified within the order *Bunyavirales*, family *Peribunyaviridae*, genus *Orthobunyavirus* [16]. This genus contains a large number of viruses, including human pathogens such as La Crosse virus (LACV) and Oropouche virus (OROV) and livestock pathogens such as Akabane virus (AKAV) and Schmallenberg virus (SBV) [17]. Orthobunyaviruses are enveloped with a negative-sense single-stranded RNA genome composed of three segments: small (S), encoding both the virus nucleoprotein and a non-structural protein (NSs); the medium (M) segment, encoding the virus glycoproteins Gn and Gc and a second non-structural protein (NSm); and the large (L) segment, encoding the virus RNA-dependent RNA polymerase [16]. The non-structural proteins interact with host cells to suppress antiviral responses and promote virus replication [18].

As a segmented virus, the opportunity for re-assortment is possible following co-infection with two different orthobunyaviruses. This mechanism is believed to have resulted in the evolution of the human pathogen Ngari virus (NRIV), which consists of the L and S segments of Bunyamwera virus (BUNV) and the M segment of BATV [19,20]. Experimental studies have shown that reassortment readily occurs in co-infected mammalian cells but not within insect-derived cells [21].

Serosurveys of cattle in Europe show extensive evidence for exposure and seroconversion to BATV [22,23,24], although evidence for BATV causing disease in animals is limited. Similarly, serosurveys in Europe also provide evidence of historic infection in human populations [25,26], although febrile disease has only been reported in humans in Africa [27]. However, the main concern for BATV acting as a human pathogen is acute hemorrhagic fever resulting from infection with NRIV in countries of East Africa [28]. Although the pathogenic mechanism for NRIV infection has not been determined, the presence of the BATV glycoproteins, the proteins that mediate cell attachment and entry, suggests that BATV could have potential to cause disease.

This review will outline in detail the vectors of BATV and the potential for this virus to cause disease in vertebrate hosts. Additionally, the current literature on BATV is inconsistent and sometimes contradictory, and therefore, this review aims to harmonize the current understanding of this virus and discuss the potential for a significant emergence in Europe.

## 2. Laboratory Detection and Diagnosis of BATV Infection

Early detections of BATV in mosquitoes were undertaken using virus isolation techniques in suckling mice [29,30]. There are now several techniques available for laboratory detection or diagnosis of BATV infection. With the advent of molecular detection techniques, reverse-transcription polymerase chain reaction (RT-PCR) has been successfully used to detect viral nucleic acid in mosquito and animal tissues. Specifically, RT-PCR has been a useful tool for high-throughput screening of large numbers of mosquitoes. Recent developments in this field include a BATV-specific RT-PCR that detects a 99 base pair region of the S segment [15] and a multiplex RT-PCR targeting BATV, Sindbis virus (SINV) and chikungunyavirus (CHIKV) that has also been successfully used for the screening of large numbers of mosquitoes [31]. The application of a multiplex assay would be particularly beneficial in regions where multiple arboviruses co-circulate, such as Italy, where BATV [14,32], CHIKV [33] and dengue virus (DENV) [34] are present, thus enabling the detection of multiple pathogens from a single source of samples.

The serological detection of neutralizing antibodies against BATV has been undertaken using techniques including a hemagglutination–inhibition (HI) test [35], a serum neutralization test (SNT) [36], immunoblotting (IB) [23], indirect immunofluorescence assay (IIFA) [23], virus neutralization test (VNT) [25] and plaque reduction neutralization test (PRNT) [37]. Early studies have demonstrated that there is little or no cross-neutralization between viruses of the Bunyamwera serogroup [38], hence, neutralization assays such as PRNT are likely to demonstrate good specificity. However, serological techniques based on virus neutralization have limitations, as they are resource heavy, require working with live virus and often take several days to complete. Therefore, although neutralization assays have utility as epidemiological tools for assessing seroprevalence, for clinical diagnosis, more rapid results would be beneficial. Recently, an in-house indirect enzyme-linked immunosorbent assay (ELISA) based on a partial recombinant BATV glycoprotein, Gc, was developed. This assay had sensitivity and specificity up to 100% and 83.7%, respectively, depending upon the species tested, which was comparable with the ‘gold standard’ SNT [36]. Therefore, for a robust clinical diagnosis, it is important that several techniques be utilized concurrently as a combination of serological (e.g., ELISA and PRNT) and molecular tests if appropriate samples are available (e.g., tissue, blood, cerebrospinal fluid). Further techniques for the detection of BATV in tissues include fluorescent in situ hybridization (FISH) [39].

## 3. Phylogenetic Analysis of BATV

**Figure 1:**
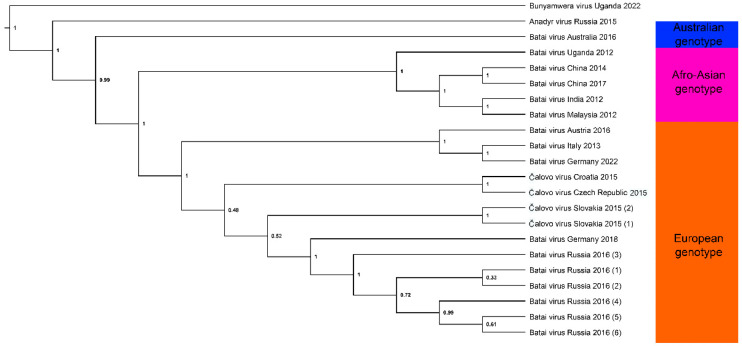
Bayesian phylogenetic analysis of BATV virus, based on a 6000 bp fragment of the L-gene from 22 sequences obtained from GenBank, with BUNV used as an outgroup. Node labels show posterior probability, and three geographically distinct genotypes are highlighted. Details of isolates used are shown in Supplementary Table S1. Isolates classed as ‘Čalovo virus’ on GenBank have retained this nomenclature in our phylogenetic analyses. Repeat detections from the same year and county were differentiated using numbers in parentheses. Briefly, sequences obtained from GenBank were aligned using MAFFT v7.471 and imported into BEAST v1.10.4 where the GTR+I substitution model and 10,000,000 Markov chain Monte Carlo generations were used to create the Bayesian phylogeny. Log files were analyzed in Tracer v1.7.1, and the resulting tree was edited in FigTree v1.4.4.

BATV has a broad geographic distribution, covering temperate and tropical regions of Asia, Africa and Europe. In addition, BATV has been recovered in multiple hosts and vectors. Therefore, understanding its phylogenetic relationships is important for understanding its emergence and persistence in a range of environmentally disparate areas and taxa. By assessing a 6000 base pair region of the L-gene from 22 BATV sequences (Supplementary Table S1) in a Bayesian phylogeny, we were able to identify three clearly delineated clades, with strong posterior probability support, within BATV (Figure 1). BUNV was used as an outgroup for the analysis, and Anadyr virus (ANADV) was also included as a novel representative of the Bunyamwera group of viruses [40]. The three clades identified are geographically distinct from one another, and our analyses support previous findings that suggest there is a European genotype and an Afro-Asian genotype [4] and add a recently identified third genotype from Australia [3].

The clustering of the African and Asian lineages supports the potential transmission of BATV from Africa to China and Malaysia through bird migration via the African–Eurasian flyway. The isolate from Australia appeared to be basal, supporting the emergence of a distinct Australian genotype, although temporal analysis with the inclusion of additional Australian sequences would be needed to confirm this. However, whether these distinct geographic lineages are infectious to different animal taxa, or whether they are vectored differentially by specific mosquito species, remains unclear.

## 4. Mosquito Vectors for BATV

The transmission of BATV by biting midges (*Culicoides* spp.) and ticks has been reported [5]; however, mosquitoes are the predominant vector group associated with BATV, with detections in a broad range of species, some of which have been shown to be competent vectors [41] (Table 1). Following the isolation of the prototype strain of BATV (AMM-2222) from *Culex gelidus* mosquitoes [1,2] and the subsequent isolations of Čalovo virus [29] and the Olyka strain [10,11] from *An. maculipennis*, further strains have been detected in *An. barbirostris* (India, strain “Chittoor” IG-20217) and *Aedes curtipes* (Sar MS-50, Sarawak) [1,10,11]. Additional mosquito species have also been shown to transmit the Chittoor strain of BATV in India such as *Cx. bitaeniorhynchus*, *An. subpictus* and *An. tessellatus* [42]. Following the detection of BATV in Slovakia, active surveillance in the last decade has recorded BATV in several countries in Europe, mainly from the zoophilic species *An. maculipennis s.l.*, but also from *An. messeae* and *An. daciae*. In addition, BATV has also been found in other species within the genera *Aedes*, subgenus *Ochlerotatus* (*Ae. vexans*, *Ae. punctor* and *Ae. communis*), *Culex* (*Cx. pipiens s.l.*, *Cx. modestus*) and *Coquillettidia* (*Cq. richiardii*). Another anopheline species, *An. claviger*, has also been recovered infected with BATV in Europe. The wide range of mosquito species in which BATV has been detected to date suggests that the number of known mosquito vector species is likely to be an underestimate. Therefore, in addition to reports of BATV detected in wild-caught mosquitoes, the emergence of BATV and related strains has led to studies assessing the vector competence of several mosquito species for BATV. These include a study demonstrating the hibernation of Čalovo virus in artificially infected females of *An. maculipennis s.l.* [43] and a study demonstrating that *Cx. quinquefasciatus* and *Cx. tritaeniorhynchus* were competent vectors of the Chittoor strain in India [44]. Furthermore, a population of *Ae. detritus* from the United Kingdom was shown to be a competent vector for a German strain of BATV [41]. Combined, these findings highlight that a range of mosquito species rather than a single genus is competent to transmit BATV. Given that these all feed on a range of different hosts, further outbreaks of BATV and the continued geographic expansion of this virus are likely.

## 5. The Role of BATV in Human and Animal Disease

BATV has the potential to infect humans, livestock animals and birds, and it can therefore be considered a threat to public and animal health. Consequently, understanding its epidemiology and future impact is of One Health importance [55,56].

### 5.1. BATV and Human Disease

BATV infection in humans is generally restricted to mild influenza-like symptoms, which can include febrile symptoms such as fever, malaise and myalgia, along with bronchopneumonia, dry and exudative pleurisy, catarrhal or follicular tonsillitis and acute gastritis [1,4]. Occasionally, symptoms may include loss of appetite, vomiting and diarrhea [4]. A 1961 report of human infections with the Čalovo strain of BATV in southern Moravia (now part of the Czech Republic) reported that human cases were generally detected from May of each year, with numbers reaching a peak in June or July before decreasing [4]; this clearly suggests a seasonal association with the abundance of mosquito vectors that peak in the warm summer months. However, many of the clinical symptoms of BATV infection are non-specific, which can complicate diagnosis in the absence of molecular testing. For example, human BATV infection in Sudan initially mis-diagnosed as malaria when based upon a febrile clinical presentation [27]. Infection with the closely related NRIV, a reassortant virus that contains the M segment of BATV, has been associated with human hemorrhagic fever, including fever with mucosal or gastrointestinal bleeding, in several African countries [13,19,20,28].

### 5.2. BATV and Animal Disease

Serological data suggest extensive exposure to BATV in European livestock [36], although to date, there have been no reports of clinical disease associated with BATV infection in ruminants in Europe [36]. Similarly, BATV has been isolated from the blood of sentinel cattle in Japan and China that were apparently healthy [5,13]. However, although BATV infection in livestock appears to be generally asymptomatic, the “Chittoor” strain of BATV was reported to cause mild illness in sheep and goats in India [6], and BATV was the cause of abortion in a small number of ruminants in Africa [7]. BATV has also been isolated from cattle in Mongolia and China that were exhibiting mild febrile disease, with loss of appetite and inability to maintain balance [5]. However, the propensity for recombination among orthobunyaviruses and the observation of more severe disease (abortion, premature birth and genetic abnormalities) in ruminants infected with the very closely related Cache Valley virus [13,57,58,59] imply that further study of the pathogenicity in mammalian hosts is warranted, in particular, the monitoring of congenital abnormalities and stillbirths in European livestock, as BATV may be missed as a differential in routine diagnosis [1]. In 2016, BATV was detected for the first time in two captive harbor seals with neurological disease in Germany [39]. Lymphohistiocytic meningoencephalomyelitis was observed in one animal, and both showed evidence of virus replication in central nervous system tissues [39]. The reason for this disease manifestation was unclear, but it suggested that the severity of disease caused by BATV may be enhanced in specific circumstances, which may include the unnatural captive environment of the seals. Additionally, it is therefore evident that severity of disease caused by BATV may be variable for different animal taxa. However, knowledge of BATV infection and disease in wild animals is limited and warrants further investigation. A recent study in Africa has demonstrated co-infection of BATV and BUNV in cattle with a history of abortion [60]. Combined, this suggests that although BATV infection alone is generally asymptomatic in livestock, there is the potential for severe disease in areas where BATV and BUNV co-circulate, which may have economic impacts in the event of an epidemic.

In terms of avian hosts, BATV has been detected in several species of birds, including carrion crow (*Corvus corone*), Eurasian coot (*Fulica atra*) and Grey Partridge (*Perdix perdix*) [61]. Antibodies against BATV have been detected in migratory birds in Slovakia [62] and in Anseriformes including graylag geese (*Anser anser*) and mallards (*Anas platyrhynchos*) in Czechoslovakia [63]. Additionally, BATV was isolated from a domestic Muscovy duck (*Cairina moschata*) flock in China where mild egg drop had been recorded [64]. To date, the role of specific animal species acting as reservoir hosts to facilitate the environmental persistence of BATV is unclear. Given that the virus has been detected in multiple insect vectors and vertebrate hosts, it is likely that several animal species could be contributing to BATV maintenance. 

## 6. The Emergence of BATV in Europe

Since the first detection of BATV (originally termed Čalovo virus) in South Slovakia in 1960, BATV infection has been sporadically detected in mosquitoes and mammalian hosts across Europe, mostly identified through serological or molecular monitoring. Details of key virus isolations, molecular detections and serological detections of BATV in Europe are shown in Table 2, which highlights the broad geographical and host species range of the virus.

Between 1960 and 1963, a serosurvey from Finland assessed neutralizing antibodies against BATV and reported prevalence rates of 0.9% in cows (n = 3190) and 0.5% in humans (n = 202). The study also assessed sera from 683 reindeer, although no neutralizing antibodies were detected. Interestingly, 172 cows within a specified area generated a 25% prevalence rate. This region was near the seashore, suggesting that an arthropod vector that may be geographically restricted to coastal locations could contribute to the natural cycle of BATV [25]. Subsequent serosurveys between 2000 and 2002 assessed wild boars (n = 93) in South Moravia (Czech Republic) and reported a prevalence of 1.1% using a PRNT and a HI test against BATV [37].

In western Europe, BATV was first detected in southwest Germany in summer 2009 in *An. maculipennis s.l.* mosquitoes; this isolate was phylogenetically similar to those from Slovakia, Ukraine and Russia [15]. The seroprevalence of BATV was subsequently assessed in southwest Germany between 2011 and 2012 and was reported to be 0.55% in healthy ruminants (n = 548), using a combination of IB, IIFA and VNT [23]. However, in eastern Germany between 2013 and 2016, BATV seroprevalence was assessed in healthy ruminants (n = 1343), and was reported to be 38.8% in goats, 44.7% in sheep and 36.4% in bovines in Saxony-Anhalt, along with 38.6% in Brandenburg (goats) and 28.4% in Saxony (goats) [24]. This was associated with detections in mosquitos during a period when *An. messeae*, *An. daciae*, *Cx. pipiens s.l.*, *Cx. modestus* and *Ae. vexans* trapped in Saxony-Anhalt and Brandenburg were shown to be infected at a prevalence of 0.58% in 4144 mosquito pools [31]. A more recent 2018 study also carried out surveillance in ruminants in Saxony-Anhalt, eastern Germany, and reported seroprevalence rates of a similar magnitude to those described between 2013 and 2016 [24], with BATV antibodies detected in sheep (16.5%), goats (18.3%) and cattle (41.4%) [36] (see Table 2). The apparent difference in seroprevalence between the earlier study from southwest Germany and the later study suggested that either the seroprevalance may have been underestimated in the earlier 2011 study or BATV had rapidly expanded in Germany, leading to an epizootic in northern Europe [41]. The BATV detected in captive harbor seals in Germany in 2016 was genetically different from the isolate from German mosquitoes and was more closely related to strains from Russia [39].

In northwest Italy, Huhtamo et al. (2013) surveyed 2589 mosquitoes in 2009, detecting BATV RNA in one pool of *An. maculipennis s.l.* [14]. Phylogenetic analysis showed that this isolate shared a high sequence identity with other European strains (Germany and Czech Republic), supporting the long-term circulation of closely related strains in Europe and a correlation between geographical location and genetic diversity [14]. This observation is supported by a study from 2014 in which the results showed a strong consensus among sequence isolates within the M, L and S segments of BATV in comparison with those isolated in Asia and Africa [9]. In 2011, the seroprevalence of BATV in Italy was reported to be 7% in healthy bovines [22], confirming the local transmission of BATV from mosquitoes to cattle. 

In comparison with animals, the antibody prevalence in humans in Europe is typically low (<1%) determined from HI tests in Sweden, Finland, Portugal, Germany, Austria, Serbia and Croatia [69], although a human seroprevalence of 32% was reported in southern Slovakia [70], which may have been related to a recent outbreak. However, the generally low antibody prevalence in humans suggests that the risk for human disease in Europe is likely low at the present time [70]. Nonetheless, there have in the past been cases of influenza-like febrile human disease arising from BATV infection in the Czech Republic [4].

**Figure 2:**
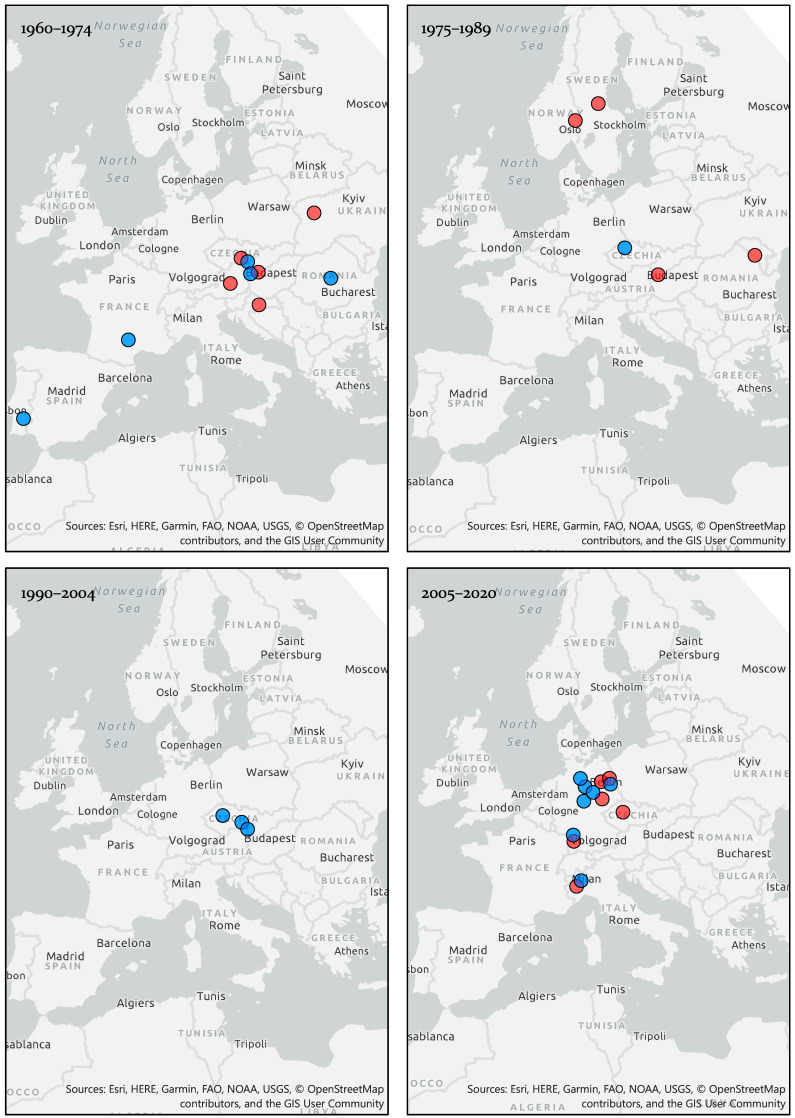
Virus isolations or molecular detections in mosquito vectors (red circles) and serological detections in mammalian hosts (blue circles) of BATV in Europe, 1960–2020. Where specific geographical locations were not available, the data point was centralized in that country or region. All geographic visualizations were created using ArcGIS Pro 2.4.

A summary of key detections of BATV in Europe, from initial detection in 1960 to the present day is shown in Figure 2.

These findings highlight that although detections have been made in several European countries, they remain sporadic in nature, most likely due to the lack of targeted surveillance. Despite this, there remains consistent BATV detection in Central Europe.

## 7. The Risk of Emergence in New Regions

The evidence to date suggests that BATV is circulating widely throughout Europe, albeit at a low to moderate level [36], and phylogenetic analysis supports the existence of a distinct genotype circulating in Europe. However, the sporadic nature of the recorded surveillance, subsequent detections and limited targeted surveillance suggest that the risk of BATV emerging in areas where it was previously undetected is likely to be underestimated. BATV has an extremely broad range of mosquito vectors, many of which are indigenous to Europe. Seroprevalence data suggest that exposure to BATV is widespread in livestock in several European countries, and there is also the possibility that BATV is already circulating in some regions but has remained undetected due to a lack of targeted surveillance. Therefore, the core components required for a substantial virus emergence are present; potentially only a slight increase in ambient temperatures would be sufficient to expand numbers of competent mosquito vector populations. The United Kingdom in particular is already beginning to experience milder winters, warmer summers and more frequent flooding events, which may enable mosquito populations to increase in abundance and remain active for longer periods of the year [71], leading to a potentially higher risk of virus emergence and transmission [72]. Additionally, the risk of mosquito-borne virus transmission is increasing in Europe due to the expanding geographical range of competent, invasive mosquito species [73], a trend that is likely to continue as average temperatures increase [74].

BATV is thought to circulate in a bird–mosquito enzootic cycle [55], although the involvement of any specific mammalian reservoir host is unclear. Early studies have suggested that cattle, sheep and swine may constitute natural BATV reservoirs [29], although further studies are necessary to definitively conclude this. However, BATV-specific neutralizing antibodies have been detected in wild birds, including house sparrows in Poland between 1995 and 1996 [75], 2.2% of house sparrows (n = 273) in the Czech Republic between 1995 and 1997 [76], and repeatedly in songbirds (Passeri) in Moravia, Czech Republic [77,78,79], with a detection rate of 12.1% in 10 species of bird (n = 198) from a Moravian fishpond habitat between 1978 and 1984 [80]. In addition, antibodies were reported against BATV in 3.6% of passerine birds caught during the autumn migration in the Krkonoše mountains, Czech Republic [78]. This strongly suggests a role for wild birds in the BATV transmission cycle, with migratory birds likely responsible for the wide geographic distribution of BATV in Europe and Asia [55], facilitating the movement of BATV into new areas potentially via the well-studied African-Eurasian flyway [60]. This is supported by our phylogenetic analyses, in which the clustering of isolates from Africa and Asia suggested the repeated transmission of BATV between the two continents.

Previous studies have suggested that BATV is unlikely to become a significant public health concern in Europe due to the relatively mild clinical symptoms and low prevalence of antibodies in humans [69]. However, there are various other factors that should be considered that suggest that BATV may be a potential threat to both human and animal health [81]. First, BATV has been shown to survive extracellularly for 30 days and can retain infectivity for up to 7 days in non-cellular medium [81]. This has potential public health implications for people exposed to infected animal blood or tissues in the event of an animal infection, as seen with the bunyavirus Rift Valley fever virus (RVFV), where exposure to virus during cattle abortion or slaughter is a major source of human infection [7]. The reasons for *ex vivo* stability are unclear but are likely to be associated with a viral mechanism to enhance transmission potential, as proposed for several other viruses, including Puumala hantavirus [82]. Additionally, the segmented genome of BATV has the potential for recombination during co-infections [18,21], suggesting that BATV could be implicated in the emergence of animal or human disease associated with orthobunyavirus infection in the future, as has been the case for NRIV. Indeed, early recombination studies concluded that genetic material was easily exchanged between viruses of the Bunyamwera serocomplex and that the *Peribunyaviridae* family of viruses as a whole have the potential for sudden and rapid variation [83]. BATV is known to co-circulate in some parts of Europe with several other mosquito-borne viruses, including Tahyna virus (TAHV) in Central Europe [1], SINV and Usutu virus (USUV) in Germany [31] and DENV [34] and CHIKV in Italy [33], giving rise to the potential for co-infections as reported for West Nile virus (WNV) and USUV in Germany [84]. There have been no reports to date of co-infection with BATV and another arbovirus in vertebrate or mosquito hosts in Europe. This could be due to current mosquito vector species assemblages, although this situation may change as additional invasive mosquito species expand their geographical range into Europe or further surveillance studies are carried out. However, previous studies have reported co-infection interference of orthobunyaviruses in both mammalian cells and mosquitoes [85,86]. Despite this, the potential for reassortment involving BATV and other mosquito-borne viruses during a co-infection warrants further investigation, particularly with viruses of the same orthobunyavirus genera that circulate in Europe, such as TAHV and Inkoo virus (INKV).

## 8. Conclusions

The evidence suggests that BATV alone is unlikely to pose a substantial risk to animal or human health in Europe at the present time. Human seroprevalence is low, and although some high seroprevalence rates have been reported in ruminants in Europe, these were not associated with clinical disease. However, clinical disease has been reported in humans due to BATV, and the reassortant NRIV, outside Europe. Additionally, neurological disease associated with BATV infection that was observed in harbor seals in Germany suggests that more severe disease is possible in animals under certain conditions, such as captivity. The continued trend for increasing summer temperatures and the frequency of flooding events in Europe may facilitate the expansion of competent mosquito populations, leading to an increased risk for mosquito-borne pathogen emergence, including BATV. Subsequently, the ability of BATV to replicate in mammalian, avian and mosquito tissues, and its segmented genome with its ability to reassort with other viruses of the Bunyamwera group, may potentially lead to the emergence of a novel reassortant virus that could constitute a One Health concern [87].

The pervasive nature of BATV in European mosquito populations and livestock suggest that BATV can be considered an emerging virus, although it currently exists at a relatively low level. However, to accurately assess the true extent of BATV in Europe and assess the risk and impact of a European BATV epidemic in the future, further targeted surveillance in both mosquitoes and sentinel animals is essential. Data on the pathogenicity of BATV in humans and livestock are also limited, and there are no data on the impact of BATV infection in wild animal populations. Studies are therefore needed to fully assess the effect of BATV infection on animal and human health, to facilitate the elucidation of the true transmission and maintenance cycles and to identify other impacts that may have previously been overlooked.

## Figures and Tables

**Table 1:** Detections of BATV and related strains detected worldwide in mosquitoes including year, location, mosquito species and virus strain (if described).

Species	Location	Virus Strain	References
*Ae. communis*	Central Sweden	85M72	[45]
*Ae. curtipes*	Sarawak (Malaysia)	Sar MS-50	[1]
*Ae. punctor*	West Ukraine	Olyka	[10,11]
*Ae. vexans*	Saxony-Anhalt, Germany; Astrakhan, Russia	*nd*	[31,46]
*An. barbirostris*	India	Chittoor IG-20217	[12,42,47]
*An. claviger*	Norway *	*nd*	[48,49]
*An. daciae*	Saxony-Anhalt, Germany	*nd*	[31]
*An. maculipennis s.l*	Čalovo, South Slovakia; Southern Moravia (Czech Republic); Austria; Croatia (former Yugoslavia)	Čalovo’ virus (strain 184)	[29,30,50,51,52,53]
*An. maculipennis.*	West Ukraine	Olyka	[10,11]
*An. maculipennis s.l.*	Zberoaia, Western Moldova; Northern Italy; Germany; Astrakhan, Russia	*nd*	[14,15,46,54]
*An. messeae*	Saxony-Anhalt, Germany	*nd*	[31]
*An. phillippinensis* (*An. philippines* in [5])	Mongolia, China	YN92-4	[5]
*An. subpictus*	Poona, India	Chittoor virus	[42]
*An. tessellatus*	Poona, India	Chittoor virus	[42]
*Cx. annulirostris*	Western Australia	K10441	[3]
*Cx. bitaeniorhynchus*	Poona, India	Chittoor virus	[42]
*Cx. gelidus*	Kuala Lumpur (Malaysia)	AMM-2222	[1,2]
*Cx. modestus*	Saxony-Anhalt, Germany	*nd*	[31]
*Cx. pipiens s.l.*	Saxony-Alhalt and Brandenburg, Germany; Astrakhan, Russia	*nd*	[31,46]
*Cq. richiardii*	Austria	Čalovo virus	[50,51]
*Culiseta *sp.**	Brandenburg, Germany	*nd*	[31]

*Ae*, *Aedes*; *An*, Anopheles; *Cq*, *Coquillettidia*; *Cx*, *Culex*; *nd*, not detailed; * identified as Bunyamwera group virus.

**Table 2:** Virus isolations or the molecular detection of BATV in mosquitoes and serological detections of BATV in mammalian hosts in Europe.

Date	Location	Species	Seroprevalence (%)	Virus Isolated (Y/N)	Reference
1960	Čalovo, south Slovakia	*An. maculipennis*	*n/a*	Y (Čalovo virus)	[29]
1960–1963	Southern Finland	Cows	0.9	N	[25]
		Cows (in defined area)	25		
		Humans	0.5		
1963	Southern Moravia (Czech Republic)	*An. maculipennis s.l.*	*n/a*	Y (Čalovo virus)	[30]
1964	South Moravia, Czech Republic	Horses	27.9	N	[65]
		Cattle	25.2		
		Pigs	17.4		
1966	Austria	*An. maculipennis s.l.*; *Coquillettidia* *richiardii*	*n/a*	Y (Čalovo virus)	[50,51]
1967	Eastern Austria	Wild boar	33.3	N	[66]
		Roe deer	70		
		Horses	100 *		
1969	Southern Portugal	Cattle and sheep	2	N	[35]
1969	Northeast Croatia (former Yugoslavia)	*An. maculipennis s.l.*	*n/a*	Y (Čalovo virus)	[52]
1971	Olkya, Western Ukraine	*An. maculipennis s.l.*; *Ae. punctor*	*n/a*	Y (Olkya strain)	[10,11]
1971	Romania	Cattle	46	N	[67]
		Sheep	41		
1975	South Slovakia	*An. maculipennis*	*n/a*	Y (Čalovo virus)	[53]
1976	Norway **	*An. claviger*	*n/a*	Y	[48]
1977	Zberoaia, Western Moldova	*An. maculipennis s.l.*	*n/a*	Y	[54]
1985	Central Sweden	*Ae. communis*	*n/a*	Y	[45]
1986–1991	Northern Bohemia and southern Moravia, Czech Republic	Roe deer	24	N	[68]
		Red deer	29		
		Fallow deer	25		
		Moufflon	50		
		Wild boar	33		
		Wild hare	13		
2002	South Moravia, Czech Republic	Wild boar	1.1	N	[37]
2002	Central Bohemia, Czech Republic	Human	1.4	N	[26]
2008	Northeast Italy	*An maculipennis s.l.*	*n/a*	N	[32]
2009	Northwest Italy	*An. maculipennis s.l.*	*n/a*	Y	[14]
2009	Southwest Germany	*An. maculipennis s.l.*	*n/a*	Y	[15]
2011	Karlsruhe, Southwest Germany	Bovines	0.55	N	[23]
2011	Northern Italy	Bovines	7.0	N	[22]
2012	Brandenburg, Germany	*Culiseta *sp.**	*n/a*	Y	[31]
2013	Brandenburg, Germany	*Cx. pipiens s.l*	*n/a*	Y	[31]
2013	Saxony-Anhalt, Germany	*An. messeae*; *An. daciae*; *Cx. pipiens s.l.*; *Cx. modestus*; *Ae. vexans*	*n/a*	Y	[31]
2013–2015	Saxony-Anhalt, Germany	Goat	38.8	N	[24]
	Brandenburg, Germany	Goat	38.6	N	
	Saxony, Germany	Goat	28.4	N	
2016	Saxony-Anhalt, Germany	Sheep	44.7	N	[24]
		Bovines	36.4		
2016	Northern Germany	Harbor seal (*Phoca vitulina*)	*nd*	Y	[39]
2018	Saxony-Anhalt, Germany	Goat	18.3	N	[36]
		Sheep	16.5		
		Cattle	41.4		

*Ae*, *Aedes*; *An*, *anopheles*; *Cx*, *Culex*; *nd*, not detailed; *n/a*, not applicable; * 2/2 horses tested were positive for antibodies against Čalovo virus; ** identified as a Bunyamwera group virus.

## Data Availability

Sequence data used for phylogenetic analysis is available from the GenBank database at: https://www.ncbi.nlm.nih.gov/nucleotide/ (accessed on 3 June 2022) (refer to accession numbers detailed in Supplementary Table S1).

## Supplementary Materials

The following supporting information can be downloaded at: https://www.mdpi.com/article/10.3390/v14091868/s1, Table S1: Accession numbers of L-gene sequences used for phylogenetic analysis of BATV.

## References

[1] Hubalek Z. (2008). Mosquito-borne viruses in Europe. Parasitol. Res..

[2] Karabatsos N. (1985). International Catalogue of Arboviruses: Including Certain Other Viruses of Vertebrates.

[3] Briese T., Williams D.T., Kapoor V., Diviney S.M., Certoma A., Wang J., Johansen C.A., Chowdhary R., Mackenzie J.S., Lipkin W.I. (2016). Analysis of Arbovirus Isolates from Australia Identifies Novel Bunyaviruses Including a Mapputta Group Virus from Western Australia That Links Gan Gan and Maprik Viruses. PLoS ONE.

[4] Sluka F. (1969). The clinical picture of the Calovo virus infection. Arboviruses of the California Complex and the Bunyamwera Group, Proceedings of the Slovak Academy of Sciences Symposium, Bratislava, Slovakia, 18–21 October 1969.

[5] Liu H., Shao X.Q., Hu B., Zhao J.J., Zhang L., Zhang H.L., Bai X., Zhang R.X., Niu D.Y., Sun Y.G. (2014). Isolation and complete nucleotide sequence of a Batai virus strain in Inner Mongolia, China. Virol. J..

[6] Pavri K.M., Singh K.R.P. (1969). Activity of Chittoor virus in India. Arboviruses of the California Complex and the Bunyamwera Group, Proceedings of the Slovak Academy of Sciences Symposium, Bratislava, Slovakia, 18–21 October 1969.

[7] Dutuze M.F. (2019). Characterization of Bunyamwera, Batai, and Ngari Viruses: Unrecognized Arboviruses of One Health Importance in Rwanda. Ph.D. Thesis.

[8] Hubalek Z. (2021). History of Arbovirus Research in the Czech Republic. Viruses.

[9] Dufkova L., Pachler K., Kilian P., Chrudimsky T., Danielova V., Ruzek D., Nowotny N. (2014). Full-length genome analysis of Calovo strains of Batai orthobunyavirus (Bunyamwera serogroup): Implications to taxonomy. Infect. Genet. Evol..

[10] Gaidamovich S.Y., Obukhova V.R., Vinograd A.I., Klisenko G.A., Melnikova E.E. (1973). Olkya-an arbovirus of the Bunyamwera group in the U.S.S.R. Acta Virol..

[11] Vinograd L.A., Gaidamovich S., Obukhova V.R., Vigovskii A.I., Emdina I.A. (1973). Study of the biological properties of the Olyka virus isolated from mosquitoes (Culicidae) in the western Ukraine. Vopr. Virusol..

[12] Yadav P.D., Sudeep A.B., Mishra A.C., Mourya D.T. (2012). Molecular characterization of Chittoor (Batai) virus isolates from India. Indian J. Med. Res..

[13] Yanase T., Kato T., Yamakawa M., Takayoshi K., Nakamura K., Kokuba T., Tsuda T. (2006). Genetic characterization of Batai virus indicates a genomic reassortment between orthobunyaviruses in nature. Arch. Virol..

[14] Huhtamo E., Lambert A.J., Costantino S., Servino L., Krizmancic L., Boldorini R., Allegrini S., Grasso I., Korhonen E.M., Vapalahti O. (2013). Isolation and full genomic characterization of Batai virus from mosquitoes, Italy 2009. J. Gen. Virol..

[15] Jost H., Bialonski A., Schmetz C., Gunther S., Becker N., Schmidt-Chanasit J. (2011). Isolation and phylogenetic analysis of Batai virus, Germany. Am. J. Trop. Med. Hyg..

[16] Hughes H.R., Adkins S., Alkhovskiy S., Beer M., Blair C., Calisher C.H., Drebot M., Lambert A.J., de Souza W.M., Marklewitz M. (2020). ICTV Virus Taxonomy Profile: Peribunyaviridae. J. Gen. Virol..

[17] Edridge A.W.D., van der Hoek L. (2020). Emerging orthobunyaviruses associated with CNS disease. PLoS Negl. Trop. Dis..

[18] Elliott R.M. (2014). Orthobunyaviruses: Recent genetic and structural insights. Nat. Rev. Microbiol..

[19] Gerrard S.R., Li L., Barrett A.D., Nichol S.T. (2004). Ngari virus is a Bunyamwera virus reassortant that can be associated with large outbreaks of hemorrhagic fever in Africa. J. Virol..

[20] Briese T., Bird B., Kapoor V., Nichol S.T., Lipkin W.I. (2006). Batai and Ngari viruses: M segment reassortment and association with severe febrile disease outbreaks in East Africa. J. Virol..

[21] Heitmann A., Gusmag F., Rathjens M.G., Maurer M., Frankze K., Schicht S., Jansen S., Schmidt-Chanasit J., Jung K., Becker S.C. (2021). Mammals preferred: Reassortment of Batai and Bunyamwera orthobunyavirus occurs in mammalian but not insect cells. Viruses.

[22] Lambert A.J., Huhtamo E., Di Fatta T., De Andrea M., Borella A., Vapalahti O., Kosoy O., Ravanini P. (2014). Serological evidence of Batai virus infections, bovines, northern Italy, 2011. Vector-Borne Zoonotic Dis..

[23] Hofmann M., Wietholter A., Blaha I., Jost H., Heinemann P., Lehmann M., Miller T., Cadar D., Yanase T., Kley N. (2015). Surveillance of Batai virus in bovines from Germany. Clin. Vaccine Immunol..

[24] Ziegler U., Groschup M.H., Wysocki P., Press F., Gehrmann B., Fast C., Gaede W., Scheuch D.E., Eiden M. (2018). Seroprevalance of Batai virus in ruminants from East Germany. Vet. Microbiol..

[25] Brummer-Korvenkontio M. (1973). Batai (Calovo) arbovirus neutralising antibodies in Finland. Ann. Med. Exp. Biol. Fenn..

[26] Hubalek Z., Zeman P., Halouzka J., Juricova Z., St’ovickova E., Balkova H., Sikutova S., Rudolf I. (2004). Antibodies against mosquito-born viruses in human population of an area of Central Bohemia affected by the flood of 2002. Epidemiol. Mikrobiol. Imunol..

[27] Nashed N.W., Olson J.G., el-Tigani A. (1993). Isolation of Batai virus (Bunyaviridae:Bunyavirus) from the blood of suspected malaria patients in Sudan. Am. J. Trop. Med. Hyg..

[28] Bowen M.D., Trappier S.G., Sanchez A.J., Meyer R.F., Goldsmith C.S., Zaki S.R., Dunster L.M., Peters C.J., Ksiazek T.G., Nichol S.T. (2001). A reassortant bunyavirus isolated from acute hemorrhagic fever cases in Kenya and Somalia. Virology.

[29] Bardos V., Cupkova E. (1962). The Calovo virus-the second virus isolated from mosquitoes in Czechoslovakia. J. Hyg. Epidemiol. Microbiol. Immunol..

[30] Smetana A., Danielova V., Kolman J.M., Malkova D., Minar J. (1967). The isolation of the Calovo virus from the mosquitoes of the group *Anopheles maculipennis* in Southern Moravia. J. Hyg. Epidemiol. Microbiol. Immunol..

[31] Scheuch D.E., Schafer M., Eiden M., Heym E.C., Ziegler U., Walther D., Schmidt-Chanasit J., Keller M., Groschup M.H., Kampen H. (2018). Detection of Usutu, Sindbis, and Batai Viruses in Mosquitoes (Diptera: Culicidae) Collected in Germany, 2011–2016. Viruses.

[32] Calzolari M., Bonilauri P., Bellini R., Caimi M., Defilippo F., Maioli G., Albieri A., Medici A., Veronesi R., Pilani R. (2010). Arboviral survey of mosquitoes in two northern Italian regions in 2007 and 2008. Vector-Borne Zoonotic Dis..

[33] Rezza G., Nicoletti L., Angelini R., Romi R., Finarelli A.C., Panning M., Cordioli P., Fortuna C., Boros S., Magurano F. (2007). Infection with chikungunya virus in Italy: An outbreak in a temperate region. Lancet.

[34] Lazzarini L., Barzon L., Foglia F., Manfrin V., Pacenti M., Pavan G., Rassu M., Capelli G., Montarsi F., Martini S. (2020). First autochthonous dengue outbreak in Italy, August 2020. Eurosurveillance.

[35] Filipe A.R., Pinto M.R. (1969). Survey for antibodies to arboviruses in serum of animals from southern Portugal. Am. J. Trop. Med. Hyg..

[36] Cichon N., Eiden M., Schulz J., Gunther A., Wysocki P., Holicki C.M., Borgwardt J., Gaede W., Groschup M.H., Ziegler U. (2021). Serological and Molecular Investigation of Batai Virus Infections in Ruminants from the State of Saxony-Anhalt, Germany, 2018. Viruses.

[37] Halouzka J., Juricova Z., Jankover J., Hubalek Z. (2008). Serologic survey of wild boars for mosquito-borne viruses in South Moravia (Czech Republic). Veterinární Med..

[38] Hunt A.R., Calisher C.H. (1979). Relationships of bunyamwera group viruses by neutralization. Am. J. Trop. Med. Hyg..

[39] Jo W.K., Pfankuche V.M., Lehmbecker A., Martina B., Rubio-Garcia A., Becker S., Kruppa J., Jung K., Klotz D., Metzger J. (2018). Association of Batai Virus Infection and Encephalitis in Harbor Seals, Germany, 2016. Emerg. Infect. Dis..

[40] Shchetinin A.M., Lvov D.K., Alkhovsky S.V., Shchelkanov M.Y., Aristova V.A., Morozova T.N., Gitelman A.K., Deryabin P.G., Botikov A.G. (2014). Complete genome analysis of the Batai virus (BATV) and the new Anadyr virus (ANADV) of the Bunyamwera group (Bunyaviridae, Orthobunyavirus) isolated in Russia. Vopr. Virusol..

[41] Hernández-Triana L.M., Folly A.J., Barrero E., Lumley S., Del Mar Fernandez de Marco M., Sewgobind S., McElhinney L.M., Fooks A.R., Johnson N. (2021). Oral susceptibility of aedine and culicine mosquitoes (Diptera: Culicidae) to Batai Orthobunyavirus. Parasit. Vectors.

[42] Singh K.R., Pavri K.M. (1966). Isolation of Chittoor virus from mosquitoes and demonstration of serological conversions in sera of domestic animals at Manjri, Poona, India. Indian J. Med. Res..

[43] Aspock H., Kunz C. (1970). Hibernation of Calovo virus in artificially infected females of *Anopheles maculipennis* Fall. Zentralbl. Bakteriol. Orig..

[44] Sudeep A.B., Shaikh N., Ghodke Y.S., Ingale V.S., Gokhale M.D. (2018). Vector competence of certain Culex and Aedes mosquitoes for the Chittoor virus, the Indian variant of the Batai virus. Can. J. Microbiol..

[45] Francy D.B., Jaenson T.G., Lundstrom J.O., Schildt E.B., Espmark A., Henriksson B., Niklasson B. (1989). Ecologic studies of mosquitoes and birds as hosts of Ockelbo virus in Sweden and isolation of Inkoo and Batai viruses from mosquitoes. Am. J. Trop. Med. Hyg..

[46] Nikiforova M.A., Kuznetsova N.A., Shchetinin A.M., Butenko A.M., Kozlova A.A., Larichev V.P., Vakalova E.V., Azarian A.R., Rubalsky O.V., Bashkina O.A. (2021). Arboviruses in the Astrakhan region of Russia for 2018 season: The development of multiplex PCR assays and analysis of mosquitoes, ticks, and human blood sera. Infect. Genet. Evol..

[47] Casals J., Whitman L. (1960). A new antigenic group of arthropod-borne viruses: The Bunyamwera group. Am. J. Trop. Med. Hyg..

[48] Traavik T., Mehl R., Wiger R. (1985). Mosquito-borne arboviruses in Norway: Further isolations and detection of antibodies to California encephalitis viruses in human, sheep and wildlife sera. J. Hyg..

[49] Becker N., Petric D., Zgomba M., Boase C., Dahl C., Lane J., Kaiser A. (2003). Mosquitoes and Their Control.

[50] Aspock H., Kunz C. (1968). Isolation of the Calovo-(=Batai-=Chitoor-)virus from *Anopheles* in Austria. Wien. Med. Wochenschr..

[51] Aspock H. (1968). Weitere Untersuchungen über die durch Stechmücken übertragenen Arboviren Österreichs Further investigations about mosquito-borne arboviruses in Austria. Zentralbl. Bakteriol. Orig..

[52] Brudnjak Z., Danielová V., Ryba J., Vesenjak-Hirjan J. (1970). Isolation of Čalovo virus from *Anopheles maculipennis sl.* mosquitoes in Yugoslavia. Folia Parasitol..

[53] Danielova V., Malkova D., Minar J., Rehse-Kupper B., Hajkova Z., Halgos J., Jedlicka L. (1978). Arbovirus isolations from mosquitoes in South Slovakia. Folia Parasitol..

[54] Chumakov M.P., Spasski A.A., Tihon E.I., Uspenskaia I.G., Konovalov Y.N. The mixed foci of arbovirus infections in Moldavia. Proceedings of the 2nd Vsesoiuznii Siezd Parazitotsenologov.

[55] Dutuze M.F., Nzayirambaho M., Mores C.N., Christofferson R.C. (2018). A Review of Bunyamwera, Batai, and Ngari Viruses: Understudied Orthobunyaviruses With Potential One Health Implications. Front. Vet. Sci..

[56] OHEJP The One Health European Joint Programme (OHEJP). https://onehealthejp.eu/about.

[57] Tsuda T. (2000). Congenital abnormalities of cattle caused by the arboviral infection. Yamaguchi J. Vet. Med..

[58] Chung S.I., Livingston C.W., Edwards J.F., Crandell R.W., Shope R.E., Shelton M.J., Collisson E.W. (1990). Evidence that Cache Valley virus induces congenital malformations in sheep. Vet. Microbiol..

[59] Rodrigues Hoffmann A., Dorniak P., Filant J., Dunlap K.A., Bazer F.W., de la Concha-Bermejillo A., Welsh C.J., Varner P., Edwards J.F. (2013). Ovine fetal immune response to Cache Valley virus infection. J. Virol..

[60] Dutuze M.F., Ingabire A., Gafarasi I., Uwituze S., Nzayirambaho M., Christofferson R.C. (2020). Identification of Bunyamwera and Possible Other Orthobunyavirus Infections and Disease in Cattle during a Rift Valley Fever Outbreak in Rwanda in 2018. Am. J. Trop. Med. Hyg..

[61] Vinograd I., Obukhova V. (1975). Isolation of arboviruses from birds in western Ukraine. Tr. Inst. Virusol..

[62] Ernek E., Kozuch O., Nosek J., Teplan J., Folk C. (1977). Arboviruses in birds captured in Slovakia. J. Hyg. Epidemiol. Microbiol. Immunol..

[63] Ernek E., Kozuch O., Nosek J., Hudec K., Folk C. (1975). Virus neutralizing antibodies to arboviruses in birds of the order Anseriformes in Czechoslovakia. Acta Virol..

[64] Zhang L., Zhang Q., Wang J., An N., Cao Y., Fu G., Hu X., Huang Y., Su J. (2017). Characterization of Batai virus isolated from a domestic Muscovy duck (*Cairina moschata*). Virus Genes.

[65] Kolman J.M. (1973). Serologic examination of some domestic animals from South Moravia on the presence of antibodies to selected arboviruses of the A, B, California and Bunyamwera groups. Folia Parasitol..

[66] Aspock H., Kunz C. (1971). Antibodies against Tahyna and Calovo viruses in wild living and domestic mammalia in the eastern Neusiedlersee area (Eastern Austria). Zentralbl. Bakteriol. Orig..

[67] Draganescu N., Gheorghiu V., Dinca A. (1971). Serologic investigations on arbovirus infections in Romania. Rev. Roum. Inframicrobiol..

[68] Hubalek Z., Juricova Z., Svobodova S., Halouzka J. (1993). A serologic survey for some bacterial and viral zoonoses in game animals in the Czech Republic. J. Wildl. Dis..

[69] Medlock J.M., Snow K.R., Leach S. (2007). Possible ecology and epidemiology of medically important mosquito-borne arboviruses in Great Britain. Epidemiol. Infect..

[70] Lundstrom J.O. (1999). Mosquito-borne viruses in western Europe: A review. J. Vector Ecol..

[71] Medlock J.M., Snow K.R., Leach S. (2005). Potential transmission of West Nile virus in the British Isles: An ecological review of candidate mosquito bridge vectors. Med. Vet. Entomol..

[72] Folly A.J., Lawson B., Lean F.Z., McCracken F., Spiro S., John S.K., Heaver J.P., Seilern-Moy K., Masters N., Hernandez-Triana L.M. (2020). Detection of Usutu virus infection in wild birds in the United Kingdom, 2020. Euro. Surveill..

[73] Schaffner F., Medlock J.M., Van Bortel W. (2013). Public health significance of invasive mosquitoes in Europe. Clin. Microbiol. Infect..

[74] EEA Global and European Temperatures. https://www.eea.europa.eu/ims/global-and-european-temperatures.

[75] Juricova Z., Pinowski J., Literak I., Hahm K.H., Romanowski J. (1998). Antibodies to alphavirus, flavivirus, and bunyavirus arboviruses in house sparrows (*Passer domesticus*) and tree sparrows (*P. montanus*) in Poland. Avian Dis..

[76] Juricová Z., Literák I., Pinowski J. (2000). Antibodies to arboviruses in house sparrows (*Passer domesticus*) in the Czech Republic. Acta Vet. Brno.

[77] Juricova Z., Hubalek Z., Halouzka J., Sikutova S. (2009). Serological examination of songbirds (Passeriformes) for mosquito-borne viruses Sindbis, Tahyna, and Batai in a south Moravian wetland (Czech Republic). Vector-Borne Zoonotic Dis..

[78] Juricová Z. (1988). Antibodies to arboviruses in wild birds caught in the Krkonoše mountains. Biológia.

[79] Juricová Z., Halouzka J., Hubálek Z. (1987). Serological examinations of birds for arboviruses in South Moravia. Biológia.

[80] Juricova Z., Hubalek Z., Halouzka J., Pellantova J., Chytil J. (1987). Haemagglutination-inhibiting antibodies against arboviruses of the families Togaviridae and Bunyaviridae in birds caught in southern Moravia, Czechoslovakia. Folia Parasitol..

[81] Dutuze M.F., Mayton E.H., Macaluso J.D., Christofferson R.C. (2021). Comparative characterization of the reassortant Orthobunyavirus Ngari with putative parental viruses, Bunyamwera and Batai: In vitro characterization and ex vivo stability. J. Gen. Virol..

[82] Kallio E.R., Klingstrom J., Gustafsson E., Manni T., Vaheri A., Henttonen H., Vapalahti O., Lundkvist A. (2006). Prolonged survival of Puumala hantavirus outside the host: Evidence for indirect transmission via the environment. J. Gen. Virol..

[83] Iroegbu C.U., Pringle C.R. (1981). Genetic interactions among viruses of the Bunyamwera complex. J. Virol..

[84] Santos P.D., Michel F., Wylezich C., Hoper D., Keller M., Holicki C.M., Szentiks C.A., Eiden M., Muluneh A., Neubauer-Juric A. (2022). Co-infections: Simultaneous detections of West Nile virus and Usutu virus in birds from Germany. Transbound. Emerg. Dis..

[85] Wernike K., Brocchi E., Beer M. (2016). Effective interference between Simbu serogroup orthobunyaviruses in mammalian cells. Vet. Microbiol..

[86] Beaty B.J., Bishop D.H., Gay M., Fuller F. (1983). Interference between bunyaviruses in Aedes triseriatus mosquitoes. Virology.

[87] Yadav P.D., Chaubal G.Y., Shete A.M., Mourya D.T. (2017). A mini-review of Bunyaviruses recorded in India. Indian J. Med. Res..

