# Supplementary material for: Batai Orthobunyavirus: An Emerging Mosquito-Borne Virus in Europe
Source: Viruses. 2022 Aug 25;14(9):1868. doi: 10.3390/v14091868 (PMC9503884; doi:10.3390/v14091868)
Supplement: Supplementary file 1 [file viruses-14-01868-s001.zip › Mansfield et al 2022 Supplementary Table S1.pdf]

Table S1. GenBank accession numbers of L-gene sequences used for phylogenetic analysis of BATV.

Virus	Accession number	Base pairs, bp	Year	Location
Batai	JX846606	6874	2012	Malaysia
Batai	JX846597	6870	2012	Malaysia
Batai	JX846600	6870	2012	India
Batai	JX846603	6870	2012	Uganda
Batai	KC168048	6870	2013	Italy
Batai	KJ187038	6870	2014	China
Anadyr	KM496335	6877	2015	Russia
Čalovo	KJ542626	6870	2015	Czech Republic
Čalovo	KJ542629	6870	2015	Croatia
Čalovo	KJ542632	6870	2015	Slovakia
Čalovo	KJ542635	6870	2015	Slovakia
Batai	KM507323	6870	2016	Austria
Batai	KU661984	6848	2016	Australia
Batai	KT313698	6714	2016	Russia
Batai	KT313701	6714	2016	Russia
Batai	KT313704	6714	2016	Russia
Batai	KT313707	6714	2016	Russia
Batai	KT313710	6714	2016	Russia
Batai	KT313713	6714	2016	Russia
Batai	KU746871	6870	2017	China
Batai	MH299974	6870	2018	Germany
Batai	NC_043580	6874	2019	Malaysia
Batai	MZ773508	6860	2022	Germany
Batai	MZ773502	6843	2022	Germany
Bunyamwera	MZ773505	6822	2022	Uganda
